# Ulcérations périnéales révélant une maladie de Crohn

**DOI:** 10.11604/pamj.2019.33.231.17761

**Published:** 2019-07-18

**Authors:** Fatima-Zahra Agharbi

**Affiliations:** 1 Hôpital Civil Tétouan, Tétouan, Maroc

**Keywords:** Crohn, ulcerations, perinée, Crohn, ulcerations, perinee

## Abstract

The whole of the lesions associated with Crohn's disease affecting the anal canal, the skin of the perineum, the bottom-rectum and the recto vaginal septum are referred to as anoperineal lesions of Crohn's disease. The main types of primary lesions include ulcerations, suppurations and stenoses. Crohn's disease-like anoperineal lesions are often suspected in patients with severe inflammatory process, peri-anal skin thickening, œdematous marisques, multiple lesions and lesions extending above pectineal line. Fistulas usually originate from an ulceration or a scar rather than the crypts of the anal canal. Elementary lesions of Crohn's disease are rarely isolated and they are, in most cases, combined lesions. The presence of a stenosis of the anal canal or of the rectum is very often associated with an inflammatory and suppurative process. Anal ulcerations are complicated with suppuration in half of the cases. We report the case of 45-year old woman treated for autoimmune thyroiditis and vitiligo who presented with knife-cut painful perineal ulcerations. The patient reported episodes of diarrhea during interview. Histological examination of skin lesions showed epitheliogigantocellular granuloma without caseous necrosis and digestive exploration suggested Crohn's disease which was confirmed with histology.

## Image en médecine

**Figure 1:**
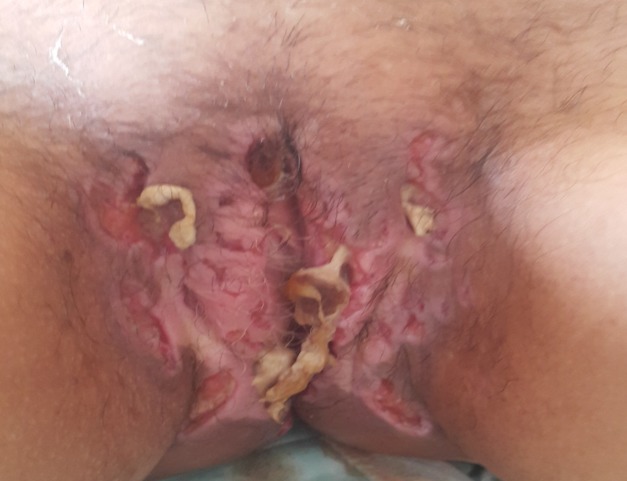
Ulcérations linéaires du périnée avec aspect coupé en couteau

On appelle lésions anopérinéales de la maladie de Crohn, l'ensemble des lésions attribuées à la maladie de Crohn qui touchent le canal anal, la peau du périnée, le bas-rectum et la cloison recto-vaginale. Les principales lésions élémentaires sont représentées par les ulcérations, les suppurations et les sténoses. La nature crohnienne des lésions anopérinéales est souvent évoquée devant un processus très inflammatoire, un épaississement de la peau péri-anale, des marisques œdémateuses, des lésions multiples, des lésions qui s'étendent au-dessus de la ligne pectinée. Les fistules prennent classiquement naissance au sein d'une ulcération ou dans une cicatrice plutôt qu'au niveau des cryptes du canal anal. Les lésions élémentaires de la maladie de Crohn sont rarement isolées et elles sont le plus souvent observées en association. La présence d'une sténose du canal anal ou du rectum est très souvent associée à un processus inflammatoire et suppuratif. Les ulcérations anales sont compliquées d'une suppuration dans la moitié des cas. Nous rapportons l'observation d'une femme de 45 ans suivie pour thyroidite auto-immune et vitiligo qui consultait pour des ulcérations périnéales douloureuses linéaires avec aspect coupées en couteau (figure 1). L'interrogatoire trouvait des épisodes de diarrhées. L'étude histologique des lésions cutanées montrait un granulome epithélio-giganto-cellualure sans nécrose casseuse et l'exploration digestive montrait un aspect évoquant une maladie de Crohn qui a été confirmé à l'histologie.

